# A multicenter, phase II trial of GC1118, a novel anti‐EGFR antibody, for recurrent glioblastoma patients with *EGFR* amplification

**DOI:** 10.1002/cam4.6213

**Published:** 2023-08-03

**Authors:** Seung Won Choi, Hyun Ae Jung, Hee‐Jin Cho, Tae Min Kim, Chul‐Kee Park, Do‐Hyun Nam, Se‐Hoon Lee

**Affiliations:** ^1^ Department of Neurosurgery School of Medicine, Sungkyunkwan University, Samsung Medical Center Seoul Republic of Korea; ^2^ Department of Medicine, Division of Hematology‐Oncology School of Medicine, Sungkyunkwan University, Samsung Medical Center Seoul Republic of Korea; ^3^ Department of Biomedical Convergence Science and Technology Kyungpook National University Daegu Republic of Korea; ^4^ Department of Internal Medicine Seoul National University Hospital, Seoul National University Cancer Research Institute, Seoul National University College of Medicine Seoul Republic of Korea; ^5^ Department of Neurosurgery Seoul National University Hospital, College of Medicine Seoul Republic of Korea; ^6^ Present address: Program for Mathematical Genomics and Department of Systems Biology Columbia University New York NY USA

**Keywords:** *EGFR*, glioblastoma, immune response, monoclonal antibody

## Abstract

**Background:**

We evaluated the therapeutic efficacy of GC1118, a novel anti‐epidermal growth factor receptor (EGFR) monoclonal antibody, in recurrent glioblastoma (GBM) patients with EGFR amplification.

**Methods:**

This study was a multicenter, open‐label, single‐arm phase II trial. Recurrent GBM patients with *EGFR* amplification were eligible: *EGFR* amplification was determined using fluorescence in situ hybridization analysis when a sample had both the EGFR/CEP7 ratio of ≥2 and a tight cluster *EGFR* signal in ≥10% of recorded cells. GC1118 was administered intravenously at a dose of 4 mg/kg once weekly. The primary endpoint was the 6‐month progression‐free survival rate (PFS6). Next‐generation sequencing was performed to investigate the molecular biomarkers related to the response to GC1118.

**Results:**

Between April 2018 and December 2020, 21 patients were enrolled in the study and received GC1118 treatment. Eighteen patients were eligible for efficacy analysis. The PFS6 was 5.6% (95% confidence interval, 0.3%–25.8%, Wilson method). The median progression‐free survival was 1.7 months (range: 28 days–7.2 months) and median overall survival was 5.7 months (range: 2–22.0 months). GC1118 was well tolerated except skin toxicities. Skin rash was the most frequent adverse event and four patients experienced Grade 3 skin‐related toxicity. Genomic analysis revealed that the immune‐related signatures were upregulated in patients with tumor regression.

**Conclusion:**

This study did not meet the primary endpoint (PFS6); however, we found that immune signatures were significantly upregulated in the tumors with regression upon GC1118 therapy, which signifies the potential of immune‐mediated antitumor efficacy of GC1118.

## 1 INTRODUCTION

Glioblastoma (GBM) is one of the most devastating malignancies with a median survival of 15 months.^1^ Most patients eventually succumb to recurrent disease despite intensive care; however, none of the current treatment has shown clinically meaningful efficacy for recurrent tumors.^2^

Many studies explored the genomic landscape of GBMs; alterations of the epidermal growth factor receptor (*EGFR*) are found in ~60% of GBM patients, including mutation and copy number amplification. These alterations usually result in constitutive activation of EGFR signaling, for example, *EGFRvIII*, the most common *EGFR* mutation in GBM, activates the tyrosine kinase without ligand binding.^3^, ^4^ Therefore, given tumor specificity and frequency, EGFR has been considered a compelling therapeutic target for GBM.

Numerous anti‐EGFR agents have been evaluated in GBMs.^5^ However, all failed to show survival benefit including EGFR tyrosine kinase inhibitors and *EGFRvIII*‐targeting peptide vaccine.^6^, ^7^ Several reasons have been suggested to account for these failures—genetic heterogeneity spanning inter‐tumor and intra‐tumor scales, signaling redundancy, the blood–brain barrier, etc.^8^, ^9^, ^10^, ^11^ Importantly, the profile of *EGFR* alteration in GBM differs from other solid cancers with sensitivity to EGFR tyrosine kinase inhibitors. *EGFR* mutations found in lung cancer usually affect the intracellular kinase domain, while *EGFR* mutations of GBMs affect the extracellular domain and are found in the context of *EGFR* amplification.

Regarding this GBM‐specific characteristic, an anti‐EGFR antibody can be an ideal option for GBMs. While previous studies with anti‐EGFR antibodies were disappointing,^12^, ^13^ the initial success of depatuxizumab‐mafodotin, an antibody‐toxin conjugate targeting the *EGFR*, was encouraging.^14^

GC1118, a novel anti‐EGFR antibody, may have selective advantages to GBMs over other anti‐EGFR antibodies; first, GC1118 has a distinct binding epitope. It recognizes a unique and critical EGFR epitope for EGF binding which does not overlap with those of other anti‐EGFR antibodies.^15^ Second, GC1118 has superior inhibitory activity against high‐affinity ligands,^15^ which are dominant in GBMs. Third, it can pass through the blood–brain barrier and even brain–tumor barrier as shown in a previous in vivo study.^16^

GC1118 has already demonstrated potential antitumor efficacy in colorectal and gastric cancers.^17^, ^18^ We also observed a comparable antitumor effect of GC1118 in in vitro and in vivo GBM models.^16^ Moreover, this efficacy was associated with high *EGFR* amplification. Based on these findings, we expected that GC1118 would be beneficial for GBM patients with *EGFR* amplification.

In this study, we aimed to evaluate the antitumor effect of GC1118 against GBM patients with *EGFR* amplification. We also conducted genomic analyses to verify the molecular correlates to clinical response.

## 2 MATERIALS AND METHODS

### 2.1 Study design and patients

This phase II, open‐label, single‐arm study was conducted at Samsung Medical Center and Seoul National University. The primary endpoint was the 6‐month progression‐free survival rate (PFS6). Secondary objectives included progression‐free survival, overall survival, radiographic response rate, and safety.

Eligible patients were ≥19 years of age with life expectancy of ≥3 months, Karnofsky performance status score ≥70, and adequate bone marrow and end‐organ function. We only included the recurrent GBM patients, who progressed following the initial Stupp regimen.^19^ *EGFR* amplification was confirmed by fluorescence in situ hybridization (FISH) analysis at each center—a sample was defined as amplified if the EGFR/CEP7 ratio was ≥2 and a tight cluster *EGFR* signal (at least 15 gene copy number) was found in ≥10% of cells. Patients who had received any EGFR targeting agents including small molecules or monoclonal antibodies were excluded.

This study (NCT03618667) was in compliance with the Declaration of Helsinki and guidelines on Good Clinical Practice. Ethics approval was obtained from local institutional review boards of each hospital (Samsung Medical Center, IRB number: 2017‐06‐111, Seoul National University Hospital, IRB number: H‐1805‐147‐948), and all patients provided informed consent.

### 2.2 Study procedures

The recommended dose for phase II trial was determined to be 4 mg/kg weekly according to the first in human trial.^20^ Patients were treated with GC1118 on Days 1, 8, 15, and 22 of a 28‐day cycle. GC1118 treatment continued up to six cycles until the disease progressed or unacceptable toxicities occurred.

Follow‐up included a weekly physical, neurologic examination, complete blood counts, and a chemical battery every 2 weeks. Brain imaging (MRI) was performed every 8 weeks. Treatment response was evaluated according to RANO criteria.^21^ We measured two diameters of each target lesion found in T1 contrast‐enhanced images; $\alpha_{i}$, the maximum length across and $\beta_{i}$, the corresponding perpendicular one to $\alpha_{i}$. We compute the sum of products of two diameters $\left(\sum_{i=1}^{N}\left(\alpha_{i}\times \beta_{i}\right)\right)$ (*N* = total number of target lesions) and compare this metric to that of baseline image. Radiographic response of existing lesion(s) was defined as follows; partial response (PR) as ≤−50%, stable disease (SD) as ≥−50% and <25%, and progression of disease (PD) as ≥25%.

Treatment was interrupted for Common Terminology Criteria for Adverse Events (AEs) version 4.03 Grade 3 drug‐related non‐hematologic toxicity (except alopecia, nausea, vomiting, and fatigue). Treatment was resumed at the physician's discretion and a dose reduction was permitted up to 2 mg/kg (1 mg/kg at each decision). Patients who experienced three or more sequential interruptions in treatment were permanently excluded from the study.

### 2.3 Statistical analysis

All patients who received at least one dose of GC1118 are eligible for safety analysis. For efficacy analysis, patients who were treated by at least one dose of GC1118 and had available tumor assessment were included.

The primary endpoint was PFS6. We applied the binomial test to compare the PFS6 of this study to previous results from the available relevant literatures (10% [range: 11%–20%]).^22^, ^23^, ^24^, ^25^, ^26^ To detect an improvement from 10% to 35%, with the power 0.8 and alpha 0.05, 18 subjects were required according to A'Hern's method.^27^ Sample size was determined to be 18 subjects, and 23 subjects were required assuming a 20% dropout rate.

All statistical analyses were performed using R version 3.6.3 (http://www.R‐project.org).^28^ Continuous variables are presented as median values and ranges or mean values with standard deviations (s.d.). *p* ≤ 0.05 was used as a threshold for statistical significance.

### 2.4 Biomarker analysis

Tumor tissue from initial diagnosis was collected and subjected to next‐generation sequencing. For the majority of patients, fresh frozen tumor tissue was available except six patients; for these patients, archival formalin‐fixed paraffin‐embedded tissue was used.

Whole‐exome sequencing data were processed accordingly as described in previous literature.^29^, ^30^, ^31^, ^32^ Briefly, somatic mutations were detected by MuTect and copy number variations were estimated using ngCGH and ABSOLUTE algorithms.^33^, ^34^ RNA‐sequencing data were processed for read counts and structural variation. We used the R package DEGseq for read count normalization (reads per kilobase of transcript per million read) and differential gene expression analysis.^35^ To detect *EGFRvIII*, GSNAP was used.^36^ For downstream analysis, pre‐ranked gene set enrichment analysis was performed using “fgsea.”^37^ We used CIBERSORTx for deconvolution analysis.^38^ A more detailed method is provided in supplementary method.

## 3 RESULTS

### 3.1 Patient characteristics

Between April 2018 and December 2020, 23 patients were screened. Two patients did not meet the inclusion criteria and 21 patients were finally enrolled and received at least one dose of GC1118 treatment. The median age was 57 years old (range: 37–71 years) and the male to female ratio was 10:11. Median Karnofsky performance status score was 70 (range: 70–90). The median number of GC1118 administrations was 7 (range: 4–22).

According to revised 2021 WHO classification, all tumors were IDH1‐wild‐type GBMs. Methylation of the MGMT promoter was identified in eight patients (38.1% [8/21]). The number of prior treatments before GC1118 treatment were as follows: one prior treatment in 66.7% (14 patients); two prior treatments in 19.0% (four patients); and three or more prior treatments in 14.3% (three patients) (Table 1). All tumors were confirmed to have EGFR amplification by FISH analysis conducted in each center.

**TABLE 1:** Baseline characteristics of study patients.

Characteristics	Intent‐to treat group (*n* = 21)	Efficacy analysis group (*n* = 18)
Age, years^a^	57 (range: 37–71)	57 (range: 37–71)
Sex		
Male	10 (47.6%)	9 (50%)
Female	11 (52.4%)	9 (50%)
KPS score at screening^a^	70 (range: 70–90)	70 (range: 70–90)
IDH1		
Wild type	21 (100%)	18 (100%)
Methylation of MGMT promoter		
Methylated	8 (38.1%)	7 (38.9%)
Unmethylated	13 (61.9%)	11 (61.1%)
Prior treatment before enrollment^b^		
1	14 (66.7%)	12 (66.7%)
2	4 (19.0%)	3 (16.7%)
≥3	3 (14.3%)	3 (16.7%)
Previous history of bevacizumab treatment		
Yes	5 (23.8%)	0 (0%)
Number of administered GC1118 doses^a^	7 (1–22)	7 (4–22)
Duration of GC1118 treatment (days)^a^	49 (28–198)	47 (0–198)

Abbreviations: KPS, Karnofsky Performance Status scale; MGMT, O^6^‐methylguanine‐DNA‐methyltransferase.

^a^ Median values are presented.

^b^ Prior treatment includes Stupp regimen, bevacizumab, low‐dose temozolomide, PCV, gamma‐knife radiosurgery, radiation therapy, and surgery other than primary surgery.

For safety analysis, we included the patients who received at least one dose of GC1118 (*n* = 21). Among these (*n* = 21), three patients discontinued GC1118 treatment after one cycle without tumor assessment: two patients refused to take further treatment after two doses of GC1118 due to a drug‐related adverse effect (skin rash); another patient experienced rapid clinical deterioration immediately after the first drug injection. Consequently, 18 patients were eligible for efficacy analysis (Figure S1).

### 3.2 Efficacy

The primary endpoint was PFS6 and only one patient was in progression‐free status at 6 months (PFS6 = 5.6% [*n* = 1/18], 95% CI, 0.3–25.8%, Wilson method).

**FIGURE 1:**
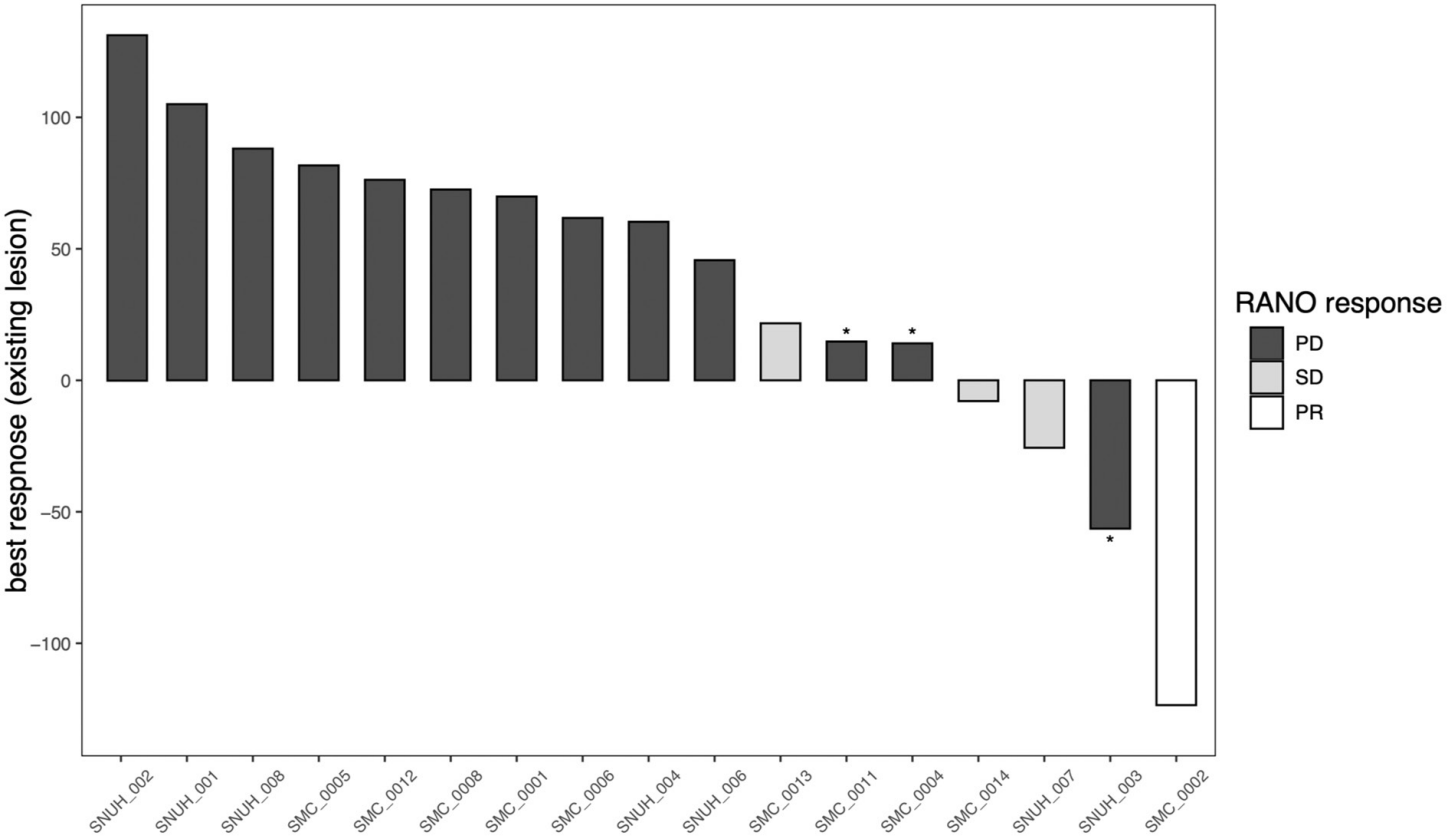
Radiographic response of existing lesion(s) following GC1118 treatment. We measured the two diameters of target lesion(s) on T1 post‐contrast images and computed products of perpendicular diameters. We compared the sum of products with baseline to evaluate the radiographic response and presented the best response in each case. Overall response was assessed by RANO criteria and presented with different colors (black‐PD; gray‐SD; white‐PR). *indicates development of new lesion including leptomeningeal seeding. All patients marked with * showed a decrease or stable state of target lesion(s); however, they were assessed as having disease progression owing to developing new lesion(s) remote from the original target lesion(s). PD, progression of disease; PR, partial response; SD, stable disease.

The median progression‐free survival was 1.7 months (range: 0.9–7.2 months) and median overall survival was 5.7 months (range: 2–22.0 months). Objective response rate was 5.6% (1 PR) and disease‐control rate was 22.2% (1 PR and 3 SD) by RANO criteria (Figure 1). We observed four cases with tumor regression—two of them (SNUH‐003 and SMC‐0002) showed a regression rate more than 50%, but one patient was defined as PD due to development of a new lesion (Figure 1).

A single patient exhibited a notable response to GC1118; patient SMC‐0002 showed regression of a contrast‐enhancing lesion following GC1118 treatment (Figure S2); however, the patient experienced a Grade 3 skin rash that necessitated dose reduction. GC1118 was reduced to 2 mg/kg, and the patient eventually developed a recurrent tumor after discontinuation of therapy. Interestingly, the epicenter of the recurrent tumor was located aside from the previously responding lesion (Figure S2).

### 3.3 Toxicity

GC1118 was generally well tolerated except for skin toxicity; none of patients experienced a Grade 4 treatment‐related AE. Treatment‐related AE profiles for all patients (*n* = 21) are summarized in Table 2.

**TABLE 2:** Treatment‐related adverse events (AEs).

AEs	Any grade	Grade 1	Grade 2	≥Grade 3
Anorexia	1 (4.8%)	0	1 (4.8%)	0
Diarrhea	2 (9.5%)	1 (4.8%)	1 (4.8%)	0
Dry skin	1 (4.8%)	1 (4.8%)	0	0
Fatigue	1 (4.8%)	1 (4.8%)	0	0
Mucositis oral	5 (23.8%)	5 (23.8%)	0	0
Nausea	1 (4.8%)	1 (4.8%)	0	0
Rash, acneiform	16 (76.2%)	3 (14.3%)	9 (42.9%)	4 (19.0%)
Rash, maculopapular	1 (4.8%)	1 (4.8%)	0	0
Vomiting	1 (4.8%)	1 (4.8%)	0	0

Skin toxicity was the most frequent AE: 76.2% of patients (16/21) experienced skin rash of at least Grade 1. Grade 2 skin toxicity was found in nine patients, and Grade 3 skin rash was noted in four patients; these four patients needed treatment interruption including dose reduction or skipped dosages.

The main cause of dose reduction was skin toxicity; GC1118 was reduced to 3 mg/kg in six patients and 2 mg/kg in two patients. Two patients (SMC‐0002 and SNUH‐008) were hospitalized because of skin rash attributed to GC1118. SMC‐0002 patient maintained the treatment with dose reduction and intermittent dose omission, while SNUH‐008 patient was dropped out due to three consecutively missed drug dosages.

### 3.4 Genomic landscape of study cohort

**FIGURE 2:**
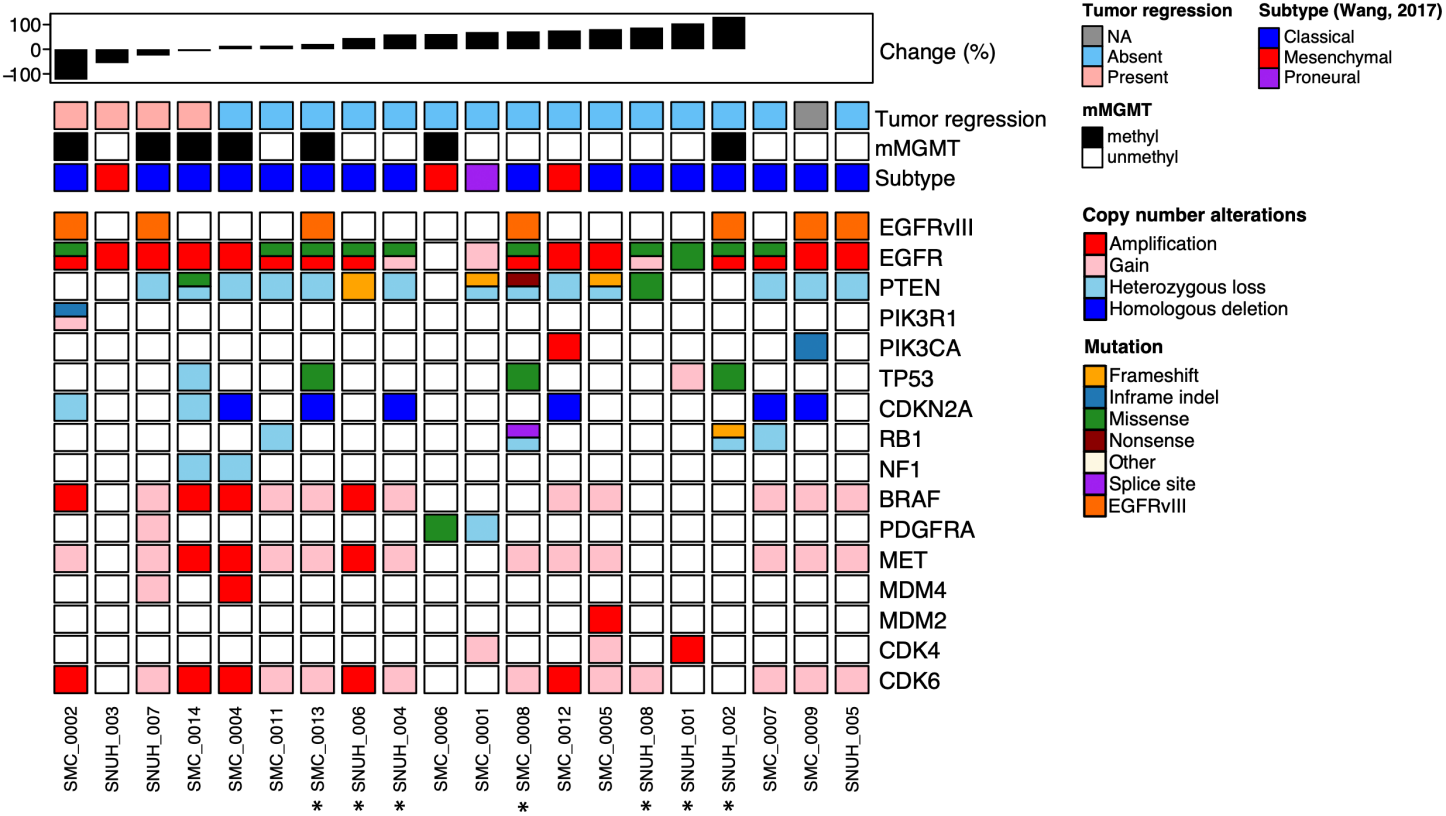
Genomic profiles of study cohort. An oncoplot depicting the genomic profiles of the study cohort. We focused on several key drivers of GBMs to analyze the mutation and copy number variation. Patients were ordered by radiographic response of existing lesion. Copy numbers derived from “GISTIC” are presented. EGFR “hot spot” variation (A289) is marked with an *. GBM, glioblastoma; EGFR, epidermal growth factor receptor; mMGMT, MGMT methylation status; NA, not available.

Genomic data were available for 20 patients, including all patients in the efficacy analysis group (Figure 2). The median of the log2 value of *EGFR* copy number was 1.7 (range: 0.2–3.7); five tumors were non‐amplified according to this analysis. As chromosome seven gain is common in GBMs, we applied ABSOLUTE to estimate the absolute copy number while adjusting the tumor purity and ploidy.^34^ Accordingly, most tumors showed high absolute copy number with two exceptional cases (mean ± s.d., 12.7 ± 3.2). This implied 90% agreement between the two methods, FISH and whole exome sequencing.

Gain‐of‐function *EGFR* mutations were found in 11 patients. Most of them were derived from the extracellular domain (81.8%, *n* = 9/11). Four patients co‐expressed *EGFRvIII* and gain‐of‐function *EGFR* mutation. A289 was the hot spot where most variations occurred (63.6%, *n* = 7/11). A previous study suggested that A289 missense mutations were associated with the clinical response to depatuxizumab‐mafodotin, potentially by modifying the receptor sensitivity.^39^ However, we did not find any significant relationship between A289 mutations status and clinical outcome in our study. In contrary, tumors with A289 mutation showed more progression (85.7%, *n* = 6/7) compared to tumors without A289 mutation (40%, *n* = 4/10), which had no prognostic implication (Figure S3).

To comprehend the molecular characteristics underlying clinical response, we investigated transcriptomic profiles. As only a single patient met the primary endpoint, we focused on whether existing tumors experienced regression during therapy. We found four patients with tumor regression during therapy (SMC‐0002, SMC‐0014, SNUH‐003, and SNUH‐007) and compared them to the remaining patients to identify the genomic signatures associated with response to GC1118.

GC1118 shows preferential inhibitory effect against high‐affinity EGFR ligands, thereby exerting a more profound therapeutic effect in a subset of tumors.^15^ We compared the mRNA expression of EGFR ligands. As anticipated, high‐affinity ligands were dominant in GBM tumors. We did not find any difference in terms of ligand expression between tumors with distinct responses (Figure S4).

We conducted pre‐ranked gene set enrichment analysis using differentially expressed genes between patients with tumor regression and without regression. Immune‐related pathways were enriched in the tumor‐regression group, while pathways involved in intercellular communication (e.g., synapses) were downregulated (Figure S5). This result was further supported by single‐sample gene set enrichment analysis: genetic signatures involved in antigen processing during the adaptive immune response were upregulated in the tumor‐regression group (Figure S5).

To understand the functional implications of enriched immune pathways in the tumor‐regression group, we deconvoluted the bulk RNA‐seq data into various cellular compositions with LM22 reference using CIBERSORTx. γδ‐T cells were significantly more abundant in the tumor‐regression group (*p*‐value = 0.008, Wilcoxon signed‐rank test). Natural killer (NK) cells, regardless of activation status, were also more frequent in responding tumors despite statistical insignificance (Figure S5).

## 4 DISCUSSION

GC1118 is a novel anti‐EGFR monoclonal antibody, which specifically binds to EGFR and inhibits the downstream cascades of the EGFR pathway.^15^ Previously, we identified *EGFR* amplification as a potential biomarker to predict the clinical response to GC1118 in an in vivo study.^16^ We adopted this result to set a phase II clinical trial, but failed to demonstrate significant clinical improvement. PFS6 was 5.6% and only one patient completed the entire course of GC1118.

The major difference between the experimental condition of the previous in vivo study and the clinical trial arises from the timely acquisition of specimens for genomic analysis. Unlike the preclinical study, we conducted genomic analysis using primary tumors while we treated the patients for their recurrent disease. Timely obtaining tumor specimens is important to make accurate molecular diagnoses, but usually impracticable in case of brain tumors.

We hypothesized that tumor evolution might contribute to this failure. We defined the temporal interval—time interval between primary tumor acquisition and GC1118 initiation—and compared it between two patients' group stratified by tumor regression. Interestingly, tumor‐regression group showed longer intervals compared to the rest (mean ± s.d., 593 ± 428 vs. 357 ± 140, respectively, *p*‐value = 0.40, Wilcoxon rank‐sum test, two‐sided).

Not only physical temporal interval, but also therapeutics may influence the evolutionary trajectory by affecting genomic integrity.^40^ We investigated the prior treatment history of patients and found that patients in the non‐regression group were far more heavily treated. Half of patients in non‐regression group had at least two distinct prior treatment regimens (42.9% [*N* = 6/14]), while all patients in tumor‐regression group were treated by standard care alone. Shortly, patients in the non‐regression group were more heavily treated during a shorter period. This may suggest innate treatment resistance or the potential of therapy‐induced tumor evolution.

Previously, many studies depicted the significance of therapeutics on the genomic evolution of malignant gliomas.^41^ Accordingly, prior intense treatment history might have a significant effect on the tumor genome, thereby boosting the shift in genomic landscape of tumors with *EGFR* dominancy, as shown in our study.

In the context of tumor evolution under therapy, *EGFR* is prone to experience clonal replacement.^40^ The mutational switching phenomenon was also highlighted in several GBM key drivers including *EGFR*. However, the functional significance of this clonal replacement in *EGFR* alteration has not been fully elucidated.

A recent study by the GLASS consortium found that the classical subtype, enriched with *EGFR* alteration, was the most plastic to subtype switching upon relapse.^42^ Loss of *EGFR* amplification was significantly associated with a shift in cell state composition, which involves mesenchymal transition. All these findings suggest that the functional dominance of *EGFR* alteration may be more vulnerable to change during treatment. However, it is noteworthy that a subset of classical tumors still maintains their transcriptomic profiles as well as genomic dominance of *EGFR*.

There are additional reasons besides tumor evolution that can account for the failure of this trial. Although we adopted the strict eligibility criteria based on FISH to select the patients with EGFR amplification, the threshold of high EGFR amplification might not be sufficient. In two cases, copy number profiles of tumors were not matched, which signifies 90% agreement of two distinct methods. This disagreement is acceptable according to literatures as whole exome sequencing normalizes copy number across the tissue sample instead of on a cell‐by‐cell basis as with FISH.^43^ However, this discrepancy may indicate the intra‐tumor heterogeneity regarding *EGFR* amplification. In other words, our criteria did not necessarily select the patients with sufficient amount of tumor cells with *EGFR* amplification to derive clinical benefit from GC1118.

Coexisting *EGFR* alterations may be potential confounders that alter the drug efficacy in this trial. A previous study found that missense mutations of the EGFR extracellular domain contributed to the receptor hypersensitivity to ligands, especially low‐affinity EGFR ligands.^13^ Many tumors in our cohort showed *EGFR* mutations, mostly affecting the extracellular domain. We found more progressive disease in tumors with an A289 missense mutation, although this trend was not translated into clinical implication. If EGFR is hypersensitized for low‐affinity ligands because of this mutation, GC1118's superior inhibitory effect against high‐affinity ligands must be weakened. We assumed that this might account for the worse treatment response observed in tumors with A289 mutation, however, we could not draw a concrete conclusion due to the limited number of cases.

Interestingly, we identified that the tumor‐regression group was enriched with immune‐related pathways by transcriptomic analysis. In a previous study, we suggested that antibody‐dependent cellular cytotoxicity might play an important role in eliciting antitumor effects of GC1118 in in vivo models.^16^

Antibody‐dependent cellular cytotoxicity driven by anti‐EGFR antibodies can induce cross talk among immune cells, especially NK cells and dendritic cells; this cross talk can prime antitumor cellular immunity.^44^, ^45^, ^46^, ^47^ A synergistic effect against tumor cells by combining anti‐EGFR antibody and immunotherapy has been validated in other solid tumors, thus, further supporting the immunologic effect of anti‐EGFR antibodies.^48^

Moreover, *EGFR* alteration has been proposed to have immunologic roles in GBMs; *EGFR* mutation governs the vascular and immune microenvironments by mediating the trans‐differentiation of glioma stem cells into pericytes.^49^ Our cases do not exactly fit in this model, but it is noteworthy that *EGFR* alteration may influence the immune landscape of gliomas and provide a stratification scheme to find patients who are sensitive to immunotherapy. A recent study also depicted the potential of *EGFR* amplification as a surrogate marker for resistance to immunotherapy in GBMs.^50^

γδ‐T cell and NK cells were upregulated in the tumor‐regression group according to deconvolution analysis. These cells are involved in the innate immune response and function as cytotoxic lymphocytes. Although γδ‐T cells have a dual effect regarding cancer progression, they can directly kill the tumor cells by diverse mechanisms including antibody‐dependent cellular cytotoxicity or indirectly induce an antitumor effect by interacting with multiple immune counterparts such as B cells, dendritic cells, and NK cells.^51^ All these findings suggest that GC1118 may exert its antitumor effect via immune‐mediated manner, at least partially.

Collectively, our study fail to show a survival benefit of GC1118 against GBMs with *EGFR* amplification. Although we compile another failure story with anti‐EGFR drug, we have learned several lessons from this study. First, as the EGFR pathway is vulnerable to tumor evolution, timely sampling is mandatory to target EGFR axis. We need to consider combinatorial treatment to overcome the emergence of new clones escaping anti‐EGFR treatment. In our study, two out of four patients with tumor regression experienced disease progression due to developing a new lesion or a recurrent lesion aside from the primary one. These findings indicate that anti‐EGFR drug alone cannot control the entire tumor cells.

We observed that immune‐related genomic signatures were upregulated in tumors with regression. This finding is similar in the context to a recent study, which suggests a hazardous effect of EGFR amplification in response to immunotherapy. These collectively suggest a potential synergism of combining GC1118 and immunotherapy. Albeit speculative, this hypothesis is worth of being explored and should be investigated in the future prospective study.

## AUTHOR CONTRIBUTIONS

**Seung Won Choi:** Formal analysis (equal); investigation (equal); visualization (equal); writing – original draft (equal); writing – review and editing (equal). **Hyun Ae Jung:** Data curation (equal); writing – original draft (equal). **Hee Jin Cho:** Formal analysis (equal); methodology (equal); visualization (equal). **Tae Min Kim:** Conceptualization (equal); project administration (equal); resources (equal). **Chul‐Kee Park:** Conceptualization (equal); project administration (equal); resources (equal). **Do‐Hyun Nam:** Conceptualization (equal); funding acquisition (equal); resources (equal); supervision (equal). **Se‐Hoon Lee:** Conceptualization (equal); funding acquisition (equal); resources (equal); supervision (equal).

## FUNDING INFORMATION

This study was supported by the GC Biopharma Corp. The funding source had no role in the design and conduct of the study; in the collection, analysis, and interpretation of the data; or in the preparation, review, or approval of the article.

## CONFLICT OF INTEREST STATEMENT

None of the authors have potential conflicts of interest with this work to declare.

## Supporting information


Data S1:

## Data Availability

The data that support the findings of this study are available on request from the corresponding author, S.H.L. The data are not publicly available due to institutional policy.

## References

[1] Koshy M , Villano JL , Dolecek TA , et al. Improved survival time trends for glioblastoma using the SEER 17 population‐based registries. J Neurooncol. 2012;107(1):207‐212. 21984115 10.1007/s11060-011-0738-7 PMC4077033

[2] Weller M , Cloughesy T , Perry JR , Wick W . Standards of care for treatment of recurrent glioblastoma—are we there yet? Neuro Oncol. 2013;15(1):4‐27. 23136223 10.1093/neuonc/nos273 PMC3534423

[3] Cancer Genome Atlas Research Network . Comprehensive genomic characterization defines human glioblastoma genes and core pathways. Nature. 2008;455(7216):1061‐1068. 18772890 10.1038/nature07385 PMC2671642

[4] Brennan CW , Verhaak RG , McKenna A , et al. The somatic genomic landscape of glioblastoma. Cell. 2013;155(2):462‐477. 24120142 10.1016/j.cell.2013.09.034 PMC3910500

[5] Roth P , Weller M . Challenges to targeting epidermal growth factor receptor in glioblastoma: escape mechanisms and combinatorial treatment strategies. Neuro Oncol. 2014;16 Suppl 8:viii14‐viii19. 25342600 10.1093/neuonc/nou222 PMC4207136

[6] Thorne AH , Zanca C , Furnari F . Epidermal growth factor receptor targeting and challenges in glioblastoma. Neuro Oncol. 2016;18(7):914‐918. 26755074 10.1093/neuonc/nov319 PMC4896544

[7] Weller M , Butowski N , Tran DD , et al. Rindopepimut with temozolomide for patients with newly diagnosed, EGFRvIII‐expressing glioblastoma (ACT IV): a randomised, double‐blind, international phase 3 trial. Lancet Oncol. 2017;18(10):1373‐1385. 28844499 10.1016/S1470-2045(17)30517-X

[8] Sottoriva A , Spiteri I , Piccirillo SG , et al. Intratumor heterogeneity in human glioblastoma reflects cancer evolutionary dynamics. Proc Natl Acad Sci U S A. 2013;110(10):4009‐4014. 23412337 10.1073/pnas.1219747110 PMC3593922

[9] Jun HJ , Acquaviva J , Chi D , et al. Acquired MET expression confers resistance to EGFR inhibition in a mouse model of glioblastoma multiforme. Oncogene. 2012;31(25):3039‐3050. 22020333 10.1038/onc.2011.474 PMC3774279

[10] Chinot OL , Wick W , Mason W , et al. Bevacizumab plus radiotherapy‐temozolomide for newly diagnosed glioblastoma. N Engl J Med. 2014;370(8):709‐722. 24552318 10.1056/NEJMoa1308345

[11] Soria JC , Ohe Y , Vansteenkiste J , et al. Osimertinib in untreated EGFR‐mutated advanced non‐small‐cell lung cancer. N Engl J Med. 2018;378(2):113‐125. 29151359 10.1056/NEJMoa1713137

[12] Neyns B , Sadones J , Joosens E , et al. Stratified phase II trial of cetuximab in patients with recurrent high‐grade glioma. Ann Oncol. 2009;20(9):1596‐1603. 19491283 10.1093/annonc/mdp032

[13] Chi AS , Cahill DP , Reardon DA , et al. Exploring predictors of response to dacomitinib in EGFR‐amplified recurrent glioblastoma. JCO Precis Oncol. 2020;4:PO.19.00295. 32923886 10.1200/PO.19.00295 PMC7446412

[14] Lassman A , Pugh S , Wang T , et al. ACTR‐21. A randomized, double‐blind, placebo‐controlled phase 3 trial of depatuxizumab mafodotin (ABT‐414) in epidermal growth factor receptor (EGFR) amplified (AMP) newly diagnosed glioblastoma (nGBM). Neuro Oncol. 2019;21(Suppl 6):vi17.

[15] Lim Y , Yoo J , Kim MS , et al. GC1118, an anti‐EGFR antibody with a distinct binding epitope and superior inhibitory activity against high‐affinity EGFR ligands. Mol Cancer Ther. 2016;15(2):251‐263. 26586721 10.1158/1535-7163.MCT-15-0679

[16] Lee K , Koo H , Kim Y , et al. Therapeutic efficacy of GC1118, a novel anti‐EGFR antibody, against glioblastoma with high EGFR amplification in patient‐derived xenografts. Cancers (Basel). 2020;12(11):3210. 33142709 10.3390/cancers12113210 PMC7693807

[17] Park JE , Jin MH , Hur M , et al. GC1118, a novel anti‐EGFR antibody, has potent KRAS mutation‐independent antitumor activity compared with cetuximab in gastric cancer. Gastric Cancer. 2019;22(5):932‐940. 30815759 10.1007/s10120-019-00943-x

[18] Lee HW , Son E , Lee K , et al. Promising therapeutic efficacy of GC1118, an anti‐EGFR antibody, against KRAS mutation‐driven colorectal cancer patient‐derived xenografts. Int J Mol Sci. 2019;20(23):5894. 31771279 10.3390/ijms20235894 PMC6928876

[19] Stupp R , Mason WP , van den Bent MJ , et al. Radiotherapy plus concomitant and adjuvant temozolomide for glioblastoma. N Engl J Med. 2005;352(10):987‐996. 15758009 10.1056/NEJMoa043330

[20] Oh DY , Lee KW , Han SW , et al. A first‐in‐human phase I study of GC1118, a novel anti‐epidermal growth factor receptor antibody, in patients with advanced solid tumors. Oncologist. 2019;24(8):1037‐e636. 31164456 10.1634/theoncologist.2019-0294 PMC6693725

[21] Wen PY , Macdonald DR , Reardon DA , et al. Updated response assessment criteria for high‐grade gliomas: response assessment in neuro‐oncology working group. J Clin Oncol. 2010;28(11):1963‐1972. 20231676 10.1200/JCO.2009.26.3541

[22] Galanis E , Anderson SK , Lafky JM , et al. Phase II study of bevacizumab in combination with sorafenib in recurrent glioblastoma (N0776): a north central cancer treatment group trial. Clin Cancer Res. 2013;19(17):4816‐4823. 23833308 10.1158/1078-0432.CCR-13-0708 PMC3869574

[23] Field KM , Simes J , Nowak AK , et al. Randomized phase 2 study of carboplatin and bevacizumab in recurrent glioblastoma. Neuro Oncol. 2015;17(11):1504‐1513. 26130744 10.1093/neuonc/nov104 PMC4648304

[24] Wick W , Puduvalli VK , Chamberlain MC , et al. Phase III study of enzastaurin compared with lomustine in the treatment of recurrent intracranial glioblastoma. J Clin Oncol. 2010;28(7):1168‐1174. 20124186 10.1200/JCO.2009.23.2595 PMC2834468

[25] Taal W , Oosterkamp HM , Walenkamp AM , et al. Single‐agent bevacizumab or lomustine versus a combination of bevacizumab plus lomustine in patients with recurrent glioblastoma (BELOB trial): a randomised controlled phase 2 trial. Lancet Oncol. 2014;15(9):943‐953. 25035291 10.1016/S1470-2045(14)70314-6

[26] Batchelor TT , Mulholland P , Neyns B , et al. Phase III randomized trial comparing the efficacy of cediranib as monotherapy, and in combination with lomustine, versus lomustine alone in patients with recurrent glioblastoma. J Clin Oncol. 2013;31(26):3212‐3218. 23940216 10.1200/JCO.2012.47.2464 PMC4021043

[27] A'Hern RP . Sample size tables for exact single‐stage phase II designs. Stat Med. 2001;20(6):859‐866. 11252008 10.1002/sim.721

[28] R. R Development Core Team . A language and environment for statistical computing. R Foundation for Statistical Computing; 2021.

[29] Li H , Durbin R . Fast and accurate short read alignment with Burrows‐Wheeler transform. Bioinformatics. 2009;25(14):1754‐1760. 19451168 10.1093/bioinformatics/btp324 PMC2705234

[30] Li H , Handsaker B , Wysoker A , et al. The sequence alignment/map format and SAMtools. Bioinformatics. 2009;25(16):2078‐2079. 19505943 10.1093/bioinformatics/btp352 PMC2723002

[31] Cibulskis K , Lawrence MS , Carter SL , et al. Sensitive detection of somatic point mutations in impure and heterogeneous cancer samples. Nat Biotechnol. 2013;31(3):213‐219. 23396013 10.1038/nbt.2514 PMC3833702

[32] Banerji S , Cibulskis K , Rangel‐Escareno C , et al. Sequence analysis of mutations and translocations across breast cancer subtypes. Nature. 2012;486(7403):405‐409. 22722202 10.1038/nature11154 PMC4148686

[33] Mermel CH , Schumacher SE , Hill B , Meyerson ML , Beroukhim R , Getz G . GISTIC2.0 facilitates sensitive and confident localization of the targets of focal somatic copy‐number alteration in human cancers. Genome Biol. 2011;12(4):R41. 21527027 10.1186/gb-2011-12-4-r41 PMC3218867

[34] Carter SL , Cibulskis K , Helman E , et al. Absolute quantification of somatic DNA alterations in human cancer. Nat Biotechnol. 2012;30(5):413‐421. 22544022 10.1038/nbt.2203 PMC4383288

[35] Wang L , Feng Z , Wang X , Wang X , Zhang X . DEGseq: an R package for identifying differentially expressed genes from RNA‐seq data. Bioinformatics. 2010;26(1):136‐138. 19855105 10.1093/bioinformatics/btp612

[36] Wu TD , Nacu S . Fast and SNP‐tolerant detection of complex variants and splicing in short reads. Bioinformatics. 2010;26(7):873‐881. 20147302 10.1093/bioinformatics/btq057 PMC2844994

[37] Korotkevich G , Sukhov V , Budin N , Shpak B , Artyomov MN , Sergushichev A . Fast gene set enrichment analysis. bioRxiv; 2019:060012.

[38] Newman AM , Steen CB , Liu CL , et al. Determining cell type abundance and expression from bulk tissues with digital cytometry. Nat Biotechnol. 2019;37(7):773‐782. 31061481 10.1038/s41587-019-0114-2 PMC6610714

[39] Hoogstrate Y , Vallentgoed W , Kros JM , et al. EGFR mutations are associated with response to depatux‐m in combination with temozolomide and result in a receptor that is hypersensitive to ligand. Neurooncol Adv. 2020;2(1):vdz051. 32642719 10.1093/noajnl/vdz051 PMC7212878

[40] Wang J , Cazzato E , Ladewig E , et al. Clonal evolution of glioblastoma under therapy. Nat Genet. 2016;48(7):768‐776. 27270107 10.1038/ng.3590 PMC5627776

[41] Barthel FP , Johnson KC , Varn FS , et al. Longitudinal molecular trajectories of diffuse glioma in adults. Nature. 2019;576(7785):112‐120. 31748746 10.1038/s41586-019-1775-1 PMC6897368

[42] Varn FS , Johnson KC , Wade TE , et al. Longitudinal analysis of diffuse glioma reveals cell state dynamics at recurrence associated with changes in genetics and the microenvironment. bioRxiv; 2021. doi:10.1101/2021.05.03.442486

[43] Lassman AB , Roberts‐Rapp L , Sokolova I , et al. Comparison of biomarker assays for EGFR: implications for precision medicine in patients with glioblastoma. Clin Cancer Res. 2019;25(11):3259‐3265. 30796037 10.1158/1078-0432.CCR-18-3034 PMC8291723

[44] Kurai J , Chikumi H , Hashimoto K , et al. Antibody‐dependent cellular cytotoxicity mediated by cetuximab against lung cancer cell lines. Clin Cancer Res. 2007;13(5):1552‐1561. 17332301 10.1158/1078-0432.CCR-06-1726

[45] Taylor RJ , Saloura V , Jain A , et al. Ex vivo antibody‐dependent cellular cytotoxicity inducibility predicts efficacy of cetuximab. Cancer Immunol Res. 2015;3(5):567‐574. 25769300 10.1158/2326-6066.CIR-14-0188 PMC4681575

[46] Lee SC , Srivastava RM , Lopez‐Albaitero A , Ferrone S , Ferris RL . Natural killer (NK): dendritic cell (DC) cross talk induced by therapeutic monoclonal antibody triggers tumor antigen‐specific T cell immunity. Immunol Res. 2011;50(2–3):248‐254. 21717064 10.1007/s12026-011-8231-0 PMC3415245

[47] Yang X , Zhang X , Mortenson ED , Radkevich‐Brown O , Wang Y , Fu YX . Cetuximab‐mediated tumor regression depends on innate and adaptive immune responses. Mol Ther. 2013;21(1):91‐100. 22990672 10.1038/mt.2012.184 PMC3538305

[48] Sacco AG , Chen R , Worden FP , et al. Pembrolizumab plus cetuximab in patients with recurrent or metastatic head and neck squamous cell carcinoma: an open‐label, multi‐arm, non‐randomised, multicentre, phase 2 trial. Lancet Oncol. 2021;22(6):883‐892. 33989559 10.1016/S1470-2045(21)00136-4 PMC12140401

[49] Segura‐Collar B , Garranzo‐Asensio M , Herranz B , et al. Tumor‐derived pericytes driven by EGFR mutations govern the vascular and immune microenvironment of gliomas. Cancer Res. 2021;81(8):2142‐2156. 33593822 10.1158/0008-5472.CAN-20-3558

[50] Friedman JS , Jun T , Rashidipour O , et al. Using EGFR amplification to stratify recurrent glioblastoma treated with immune checkpoint inhibitors. Cancer Immunol Immunother. 2023;72:1893‐1901. 36707424 10.1007/s00262-023-03381-y PMC10992363

[51] Zhao Y , Niu C , Cui J . Gamma‐delta (gammadelta) T cells: friend or foe in cancer development? J Transl Med. 2018;16(1):3. 29316940 10.1186/s12967-017-1378-2 PMC5761189

