# Supplementary material for: A multicenter, phase II trial of GC1118, a novel anti‐EGFR antibody, for recurrent glioblastoma patients with EGFR amplification
Source: Cancer Med. 2023 Aug 3;12(15):15788–96. doi: 10.1002/cam4.6213 (PMC10469652; doi:10.1002/cam4.6213)

**Supplementary Information**

**Supplementary Methods**

**Biomarker analysis**

Tumor specimens (fresh frozen tissue or unstained slides from formalin-fixed paraffin-embedded tissue blocks) were collected and subjected to next-generation sequencing for further analysis. Genomic DNA and mRNA were extracted using the DNeasy kit and the RNeasy kit (Qiagen), respectively.

For whole-exome sequencing data, the sequenced reads in the FASTQ files were aligned to the human genome assembly (hg19) using Burrows-Wheeler aligner.(1) The initial alignment BAM files were preprocessed for sorting, removing duplicate reads, realigning reads around potential small indels, and recalibrating base quality score using SAMtools.(2) MuTect and Somatic IndelDetector were used to make high-confidence predictions on somatic mutations from neoplastic and non-neoplastic tissue pairs. (3, 4) Variant Effector Predictor was used to annotate somatic mutations.(5) To estimate copy number variations (CNVs), the processed bam files were subjected to ngCGH algorithm (python package) which provides the median-centered log2 ratio between tumor and normal per each segment. Then, we calculated the CNV of each gene based on the resulting segment file with GISTIC2.0.(6) Also, ABSOLUTE algorithm generated the absolute copy number values using mutational profiles and CNV segment information for each sample.(7)

For all samples, RNA-seq libraries were prepared using the Illumina TruSeq RNA Sample Prep kit. RNA-seq data were analyzed for two types of information: mRNA expression level and structure variations. For analysis of the mRNA expression level, sequenced reads in FASTQ files were trimmed to include only 30 nucleotides from the 5′ end of each read. The trimmed reads were mapped on hg19 using GSNAP version 2012-12-20.(8) The resulting alignment SAM files were sorted and summarized into BED files using SAMtools and bedTools (bamToBed version 2.16.2).(2) The BED files were used to calculate reads per kilobase of transcript per million reads (RPKM) and mapped values for each gene by using the R package DEGseq and the RefSeq gene annotations.(9) For exon skipping analysis to detect EGFR vIII, GSNAP was used to perform paired-end-mode mapping of the reads on the pair of gene sequences involved in the exon skipping without allowing any mismatch, indel, or splicing.(8) The identified paired-end reads were mapped on hg19 by BLAT to reveal break-/fusion-points.

To explore the transcriptomic profiles associated with tumor regression, we performed pre-ranked gene set enrichment analysis (GSEA) using ‘fgsea’ package in R.(10) First, we conducted differential expression analysis between two groups, tumors with regression versus tumors without regression (‘DESeq2’ package in R), and generated the ranked gene list.(11) A total of 118 differentially expressed genes (FDR < 0.1) were ranked by statistics and then subjected to pre-ranked GSEA. We searched curated gene sets from MSigDB v7.4 (Biocarta, KEGG, Reactome, GO, and Hallmark) to find the most relevant genomic signatures associated with tumor regression. To validate the results from pre-ranked GSEA, we compared the single-sample GSEA scores of two groups using the two-sided Wilcoxon rank sum test.(12)

To infer the composition of distinct cell types, we deconvoluted our bulk sample RNAseq data using CIBERSORTx.(13) We used the publicly available signature matrix LM22 to compute distinct immune cell proportions.(14)

**References**

1. Li H, Durbin R. Fast and accurate short read alignment with Burrows-Wheeler transform. Bioinformatics. 2009;25(14):1754-60.

2. Li H, Handsaker B, Wysoker A, Fennell T, Ruan J, Homer N, et al. The Sequence Alignment/Map format and SAMtools. Bioinformatics. 2009;25(16):2078-9.

3. Banerji S, Cibulskis K, Rangel-Escareno C, Brown KK, Carter SL, Frederick AM, et al. Sequence analysis of mutations and translocations across breast cancer subtypes. Nature. 2012;486(7403):405-9.

4. Cibulskis K, Lawrence MS, Carter SL, Sivachenko A, Jaffe D, Sougnez C, et al. Sensitive detection of somatic point mutations in impure and heterogeneous cancer samples. Nat Biotechnol. 2013;31(3):213-9.

5. McLaren W, Gil L, Hunt SE, Riat HS, Ritchie GR, Thormann A, et al. The Ensembl Variant Effect Predictor. Genome Biol. 2016;17(1):122.

6. Mermel CH, Schumacher SE, Hill B, Meyerson ML, Beroukhim R, Getz G. GISTIC2.0 facilitates sensitive and confident localization of the targets of focal somatic copy-number alteration in human cancers. Genome Biol. 2011;12(4):R41.

7. Carter SL, Cibulskis K, Helman E, McKenna A, Shen H, Zack T, et al. Absolute quantification of somatic DNA alterations in human cancer. Nat Biotechnol. 2012;30(5):413-21.

8. Wu TD, Nacu S. Fast and SNP-tolerant detection of complex variants and splicing in short reads. Bioinformatics. 2010;26(7):873-81.

9. Wang L, Feng Z, Wang X, Wang X, Zhang X. DEGseq: an R package for identifying differentially expressed genes from RNA-seq data. Bioinformatics. 2010;26(1):136-8.

10. Korotkevich G SV, Sergushichev A Fast gene set enrichment analysis. 2019.

11. Love MI HW, Anders S. Moderated estimation of fold change and dispersion for RNA-seq data with DESeq2. Genome Biology. 2014(15):550.

12. Hanzelmann S, Castelo R, Guinney J. GSVA: gene set variation analysis for microarray and RNA-seq data. BMC Bioinformatics. 2013;14:7.

13. Newman AM, Steen CB, Liu CL, Gentles AJ, Chaudhuri AA, Scherer F, et al. Determining cell type abundance and expression from bulk tissues with digital cytometry. Nat Biotechnol. 2019;37(7):773-82.

14. Newman AM, Liu CL, Green MR, Gentles AJ, Feng W, Xu Y, et al. Robust enumeration of cell subsets from tissue expression profiles. Nat Methods. 2015;12(5):453-7.

**Supplementary Figures & Legends**

**Supplementary Figure S1**. CONSORT diagram of study cohort


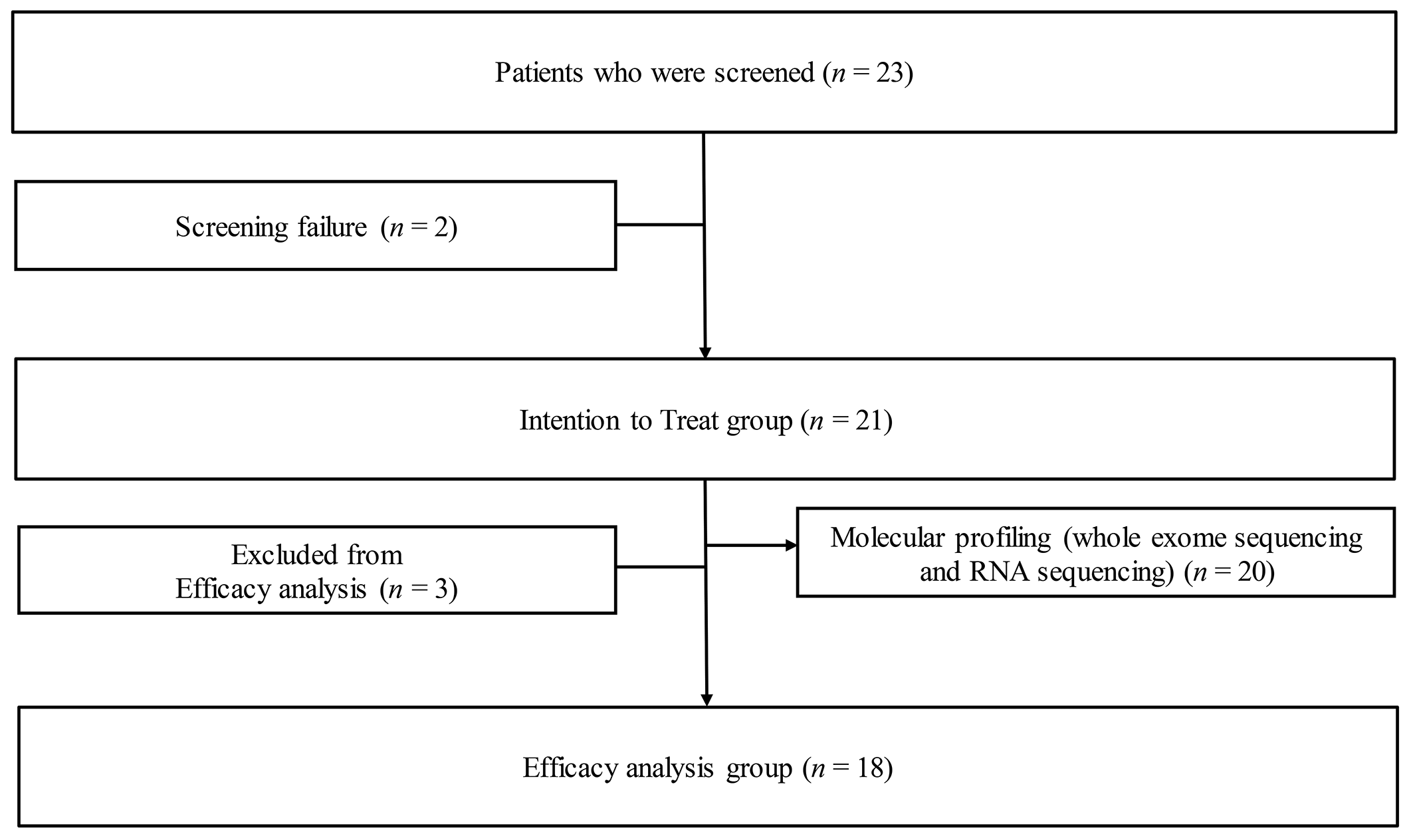


**Supplementary Figure S2.** Case illustration (SMC-0002). Representative MR images of SMC-0002 patient are presented in chronological order. Tumor showed consecutive regression during therapy. He suffered Gr 2 - 3 skin toxicity that interrupted the dosing schedule and resulted in dose reduction (red arrow). After completion of entire GC1118 cycles, he refused to take further treatment. Follow-up MR images (D217) taken at 3 weeks after GC1118 discontinuation demonstrated recurrent tumor; epicenter of recurrent tumor (bottom) was apart from the primary tumor (top), suggesting the marginal recurrence.


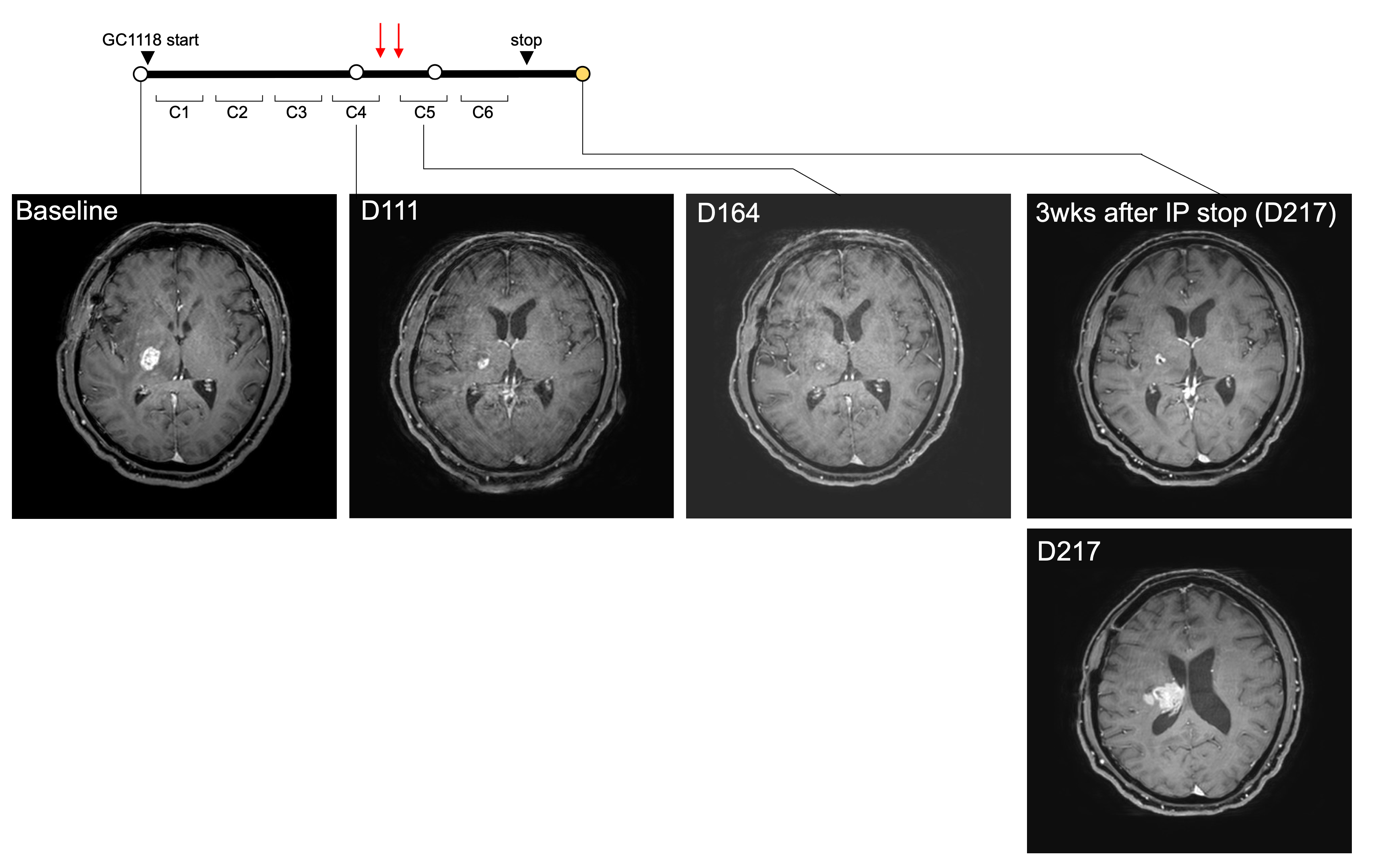


**Supplementary Figure S3.** Clinical response to GC1118 in tumors stratified by A289 mutation status

**Supplementary Figure S4.** Bar plots of mRNA expression of EGFR ligands.


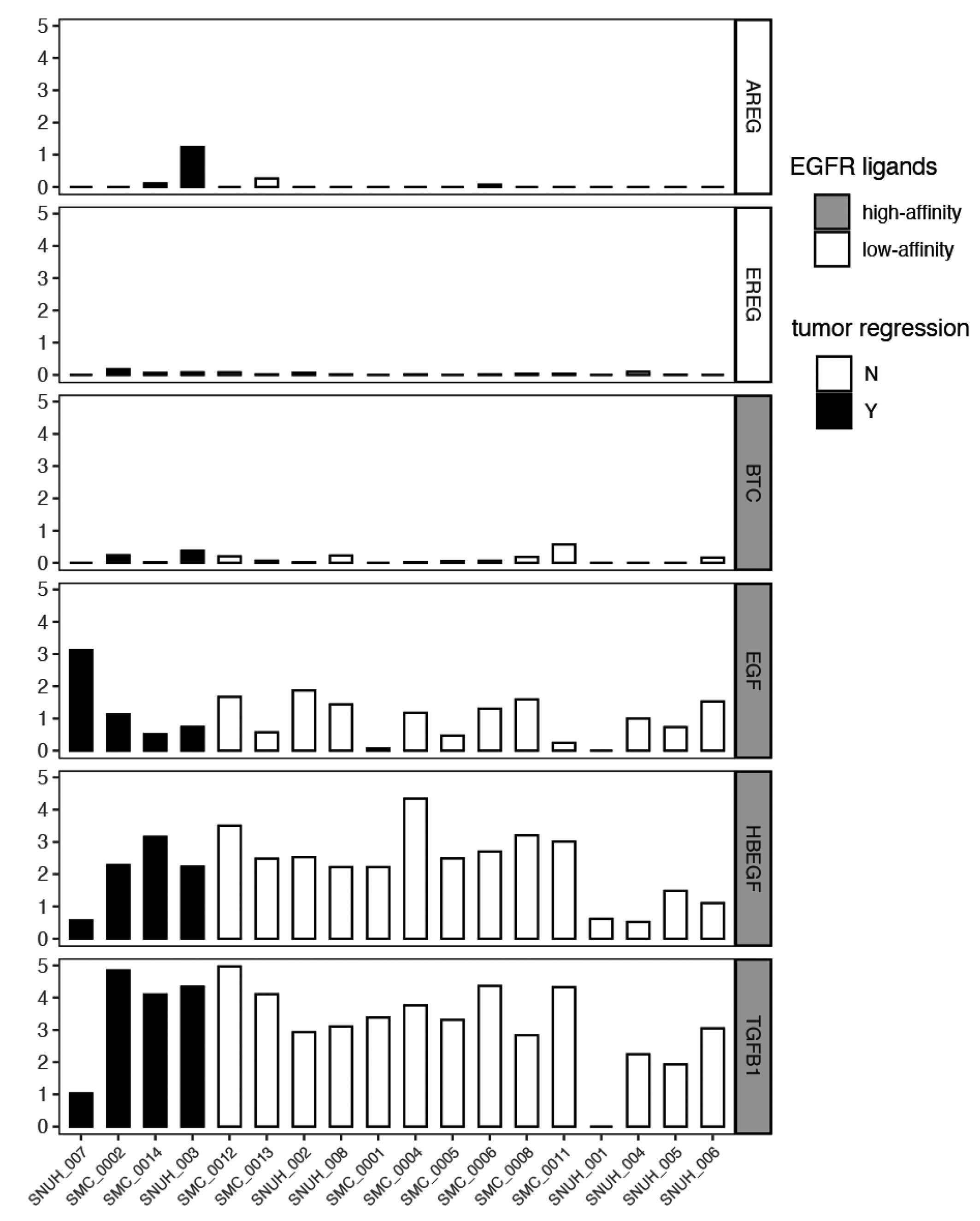


**Supplementary Figure S5.** Genomic signatures associated with clinical response to GC1118 treatment. (A) A heatmap depicting gene sets that were significantly enriched in the tumor-regression group (FDR < 0.05) according to pre-ranked gene set enrichment analysis. Abbreviations: *NES, normalized enrichment score. (B) Gene set enrichment analysis plots of most upregulated (top) and downregulated (bottom) gene sets in tumor-regression group. (C) A volcano plot depicting the comparison of single sample gene set enrichment analysis (ssGSEA) scores between tumor-regression and non-regression group. Gene sets with *P*-value < 0.01 are marked with red color. Genomic signatures involved in antigen processing and presentation were significantly highlighted in tumor-regression group (black circle). Bar plots presenting ssGSEA scores of these signatures are shown at the bottom. (D) Immune cell proportions deconvoluted from bulk RNAseq data are presented. Abbreviations: *FC, fold change


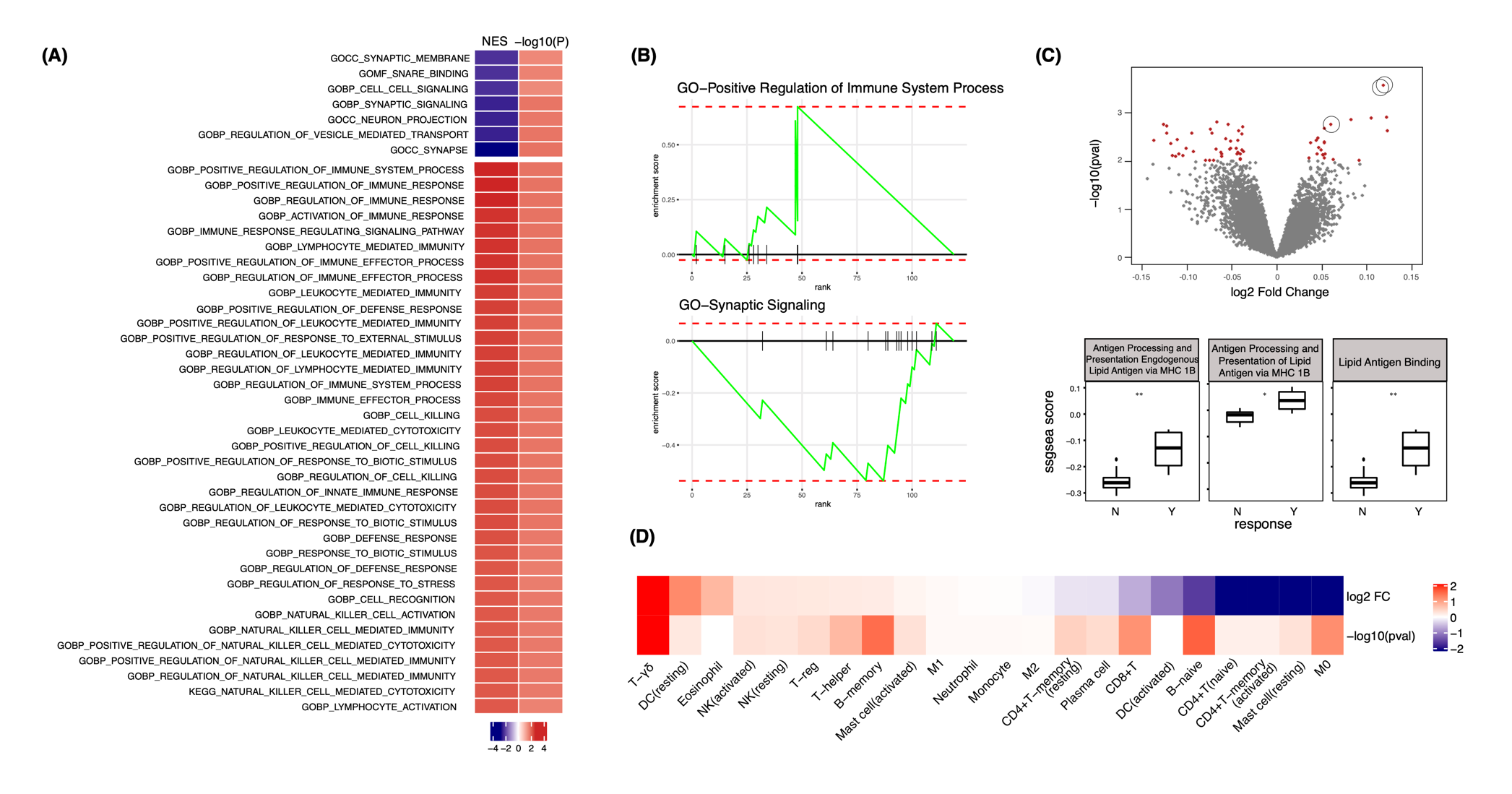

Supplement: Supplementary file 1 — Data S1: [file CAM4-12-15788-s001.docx]
